# Interstitial cells of Cajal: clinical relevance in pediatric gastrointestinal motility disorders

**DOI:** 10.1007/s00383-023-05467-1

**Published:** 2023-04-27

**Authors:** Florian Friedmacher, Udo Rolle

**Affiliations:** Department of Paediatric Surgery and Paediatric Urology, University Hospital Frankfurt, Goethe University Frankfurt, Theodor-Stern-Kai 7, 60590 Frankfurt, Germany

**Keywords:** Interstitial cells of Cajal, c-Kit, Anoctamin-1, Smooth muscle cells, Enteric nervous system, Hirschsprung disease

## Abstract

Interstitial cells of Cajal (ICCs) are pacemaker cells of gastrointestinal motility that generate and transmit electrical slow waves to smooth muscle cells in the gut wall, thus inducing phasic contractions and coordinated peristalsis. Traditionally, tyrosine-protein kinase Kit (c-kit), also known as CD117 or mast/stem cell growth factor receptor, has been used as the primary marker of ICCs in pathology specimens. More recently, the Ca^2+^-activated chloride channel, anoctamin-1, has been introduced as a more specific marker of ICCs. Over the years, various gastrointestinal motility disorders have been described in infants and young children in which symptoms of functional bowel obstruction arise from ICC-related neuromuscular dysfunction of the colon and rectum. The current article provides a comprehensive overview of the embryonic origin, distribution, and functions of ICCs, while also illustrating the absence or deficiency of ICCs in pediatric patients with Hirschsprung disease intestinal neuronal dysplasia, isolated hypoganglionosis, internal anal sphincter achalasia, and congenital smooth muscle cell disorders such as megacystis microcolon intestinal hypoperistalsis syndrome.

## Introduction

Interstitial cells of Cajal (ICCs) were first described in 1893 by the Spanish histopathologist and Nobel Prize laureate Santiago Ramón y Cajal as “*fibroblast-like cells in the muscularis externa and villous stroma of the gastrointestinal tract*” [1]. Later, light and electron microscopy studies demonstrated that ICCs are neither neurons nor Schwann cells [2–4], forming a unique class of cells that are distinct from the enteric nervous system (ENS) [5]. In the 1990s, the tyrosine-protein kinase Kit (c-kit), also known as CD117 or mast/stem cell growth factor receptor, has been identified as the primary marker of ICCs in pathology specimens [6, 7]. However, while loss of c-kit positivity is not necessarily indicative of loss of ICCs, it also follows that normal levels of c-kit positivity are not automatically suggestive of normal ICC distribution [8, 9]. This, in addition to the finding that c-kit also labels mast cells in the circular muscle layer [10], has led recently to the introduction of the Ca^2+^-activated chloride channel anoctamin-1 (Ano1) as more specific marker of ICCs [11–13].

Today, it is well established that ICCs are distributed throughout the entire alimentary tract, from the upper esophageal sphincter to the internal sphincter of the anus [14–16]. ICCs are located between the nerve endings of motor neurons and smooth muscle cells, modulating inhibitory and excitatory signals from the ENS [9, 17]. They play a major role in gastrointestinal motility by generating slow-wave electrical activity, which propagates throughout the smooth muscle layers of the gut, giving rise to peristaltic waves [8]. In 2013, it was reported that allotransplantation of ICCs could not only populate tissues but also establishes functional pacemaker activity, where they originally were absent [18]. Thus, further research in this field may provide the basis for a therapeutic treatment of gastrointestinal motility disorders in patients, where ICC networks have been disrupted or lost, for example due to genetic defects, pathophysiological insults or natural aging processes.

### Embryonic origin and development of ICCs

In contrast to Cajal’s opinion, ICCs develop independently of neural crest-derived enteric neurons and glia, and originate mainly from c-kit-positive mesenchymal precursor cells [19–22]. Furthermore, it has been shown that the normal development of ICCs depends on the expression of c-kit [6, 7]. *Kit* signaling is essential for both the development and maintenance of functional ICCs in the embryonic gastrointestinal tract, with precursor ICCs expressing c-kit as early as embryonic day 11 (E11) in mice [20]. At E12, c-kit-positive cell clusters were found in the periphery of developing murine small intestine, just under the serosal surface. C-kit-positive cells occur from E15 onwards peripheral to developing myenteric ganglia [23]. The late gestational time between E15 and E18 seems to be a critical period during ICC development as the c-kit-positive precursors begin to develop toward a functional ICC phenotype. The pharmacological or genetic blockade of *Kit* signaling during late gestation results in the failure of ICC networks and pacemaker function to develop in the small intestine. However, the ICC network appears to have a certain plasticity, allowing for restorative changes and redevelopment of functional ICCs [21].

Various studies have investigated the fetal and postnatal development of ICCs in the human gastrointestinal tract, demonstrating c-kit-positive cells in the stomach from 9.5 weeks of gestation and in the small and large bowel from 12 to 13 weeks [24–26]. ICCs also undergo significant changes postnatally. The number of ICC cell bodies and volume of ICC within the human stomach and colon decrease with age at a rate of 13% per decade, with no differences according to sex or location in the gastrointestinal tract [27].

### Functions and distribution of ICCs

Over a century ago, the Scottish anatomist Sir Arthur Keith had already suggested that ICCs might act as pacemaker cells of gastrointestinal motility, coordinating phasic contractile activity [28]. Later on, electron microscopical studies have shown a close association of ICCs with nerve terminals and gap junctions within smooth muscle cells [5, 29]. Today, numerous gastrointestinal functions are known that are affected by ICCs (e.g., generating and active propagation of electrical slow waves, depolarization into adjacent smooth musculature, etc.).

Extensive morphological and electrophysiological research has revealed multiple complex functions of ICCs (Box 1).

Several subtypes of ICCs have been distinguished according to their distinct distribution patterns and morphological features within the anatomical layers of the gastrointestinal tract [34]. Each type of ICCs is determined by the structure of their adjacent smooth muscle layer, their relation to neighboring nerve plexuses and the density of their connections with other ICCs (Table 1).

**Table 1:** Summary of ICC subtypes

Tissue layer	Distribution pattern and morphological features of ICC network
Submucosa and submucosal plexus	ICCs are located at the interface between the submucosal layer and the innermost circular muscle layer of stomach and colon [35–39]. These multipolar cells form a loose network via their secondary processes [6, 40]
Circular muscle	ICCs are bipolar and orientated along the surrounding muscle cells. Their distribution and density vary considerably within the gastrointestinal tract. They are sparsely found in the small bowel without forming a network, whereas they are strongly expressed along the nerve bundles of stomach and colon [33] (Fig. 1). Within the deep muscular plexus of the small bowel, also multipolar ICCs are found along the inner portion of the circular muscle layer in close proximity to nerve bundles [41, 42]
Myenteric plexus	ICCs show here the greatest density, being multipolar cells with 3–5 primary processes that are connected to each other and to neighboring structures (Fig. 2). They form a dense network in the small bowel and are less dense in stomach and colon [33]. ICCs also project deep into the circular muscle layer via septa that separate the circular muscle into bundles. Their projections may provide a pathway through which slow waves are propagated into the depth of the circular muscle layer, which is most likely the case in animals with thicker muscle layers [43]
Longitudinal muscle	ICCs are bipolar and similar to those in the circular muscle layer, but less numerous [33] (Fig. 3)
Subserosa	ICCs comprise a group of stellate cells in small bowel and colon of mice and guinea pigs [6, 44]

Box 1 functions of ICCs

1. Pacemaker cells that actively propagate electrical slow waves to gastrointestinal smooth muscle cells [30].
2. Mediators of both inhibitory and excitatory motor neurotransmission from the ENS [7, 30, 31]
3. Non-neural stretch receptors in gastrointestinal muscle, affecting both smooth muscle excitability and slow-wave frequency [32]
4. Formation of a network with close associations to the intramuscular terminals of vagal afferents and may also have a role in afferent signaling [33]

### Identification and visualization of ICCs

**Fig. 1:**
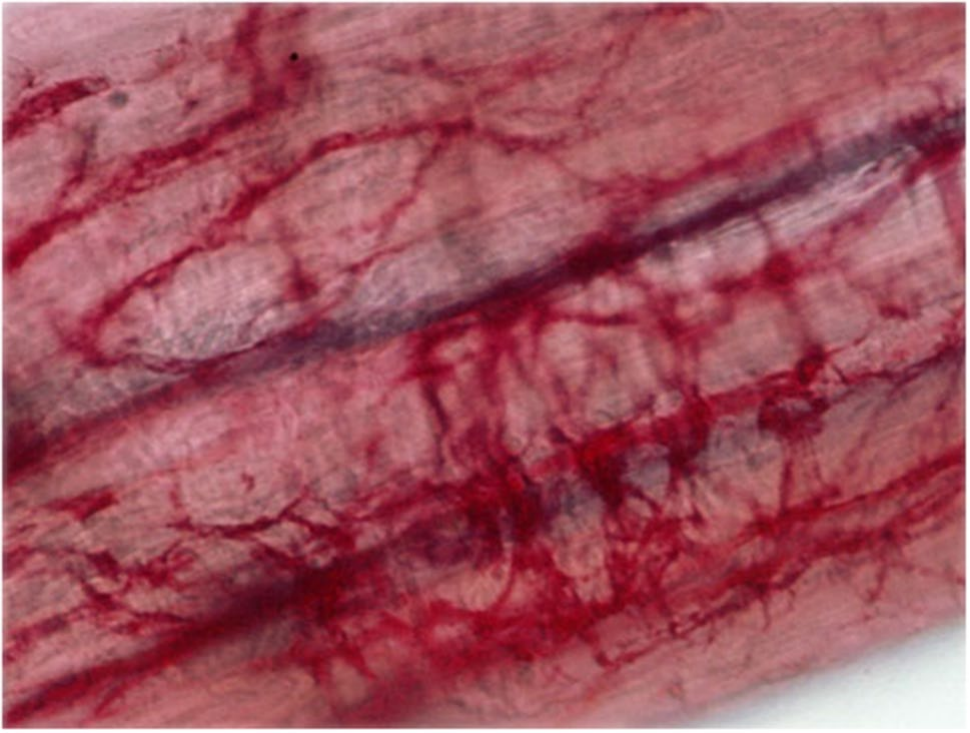
Whole-mount preparation of circular muscle of human colon, nerve fibers stained with NADPH-diaphorase (blue) and muscular ICCs stained with anti-c-kit immunohistochemistry (red)

**Fig. 2:**
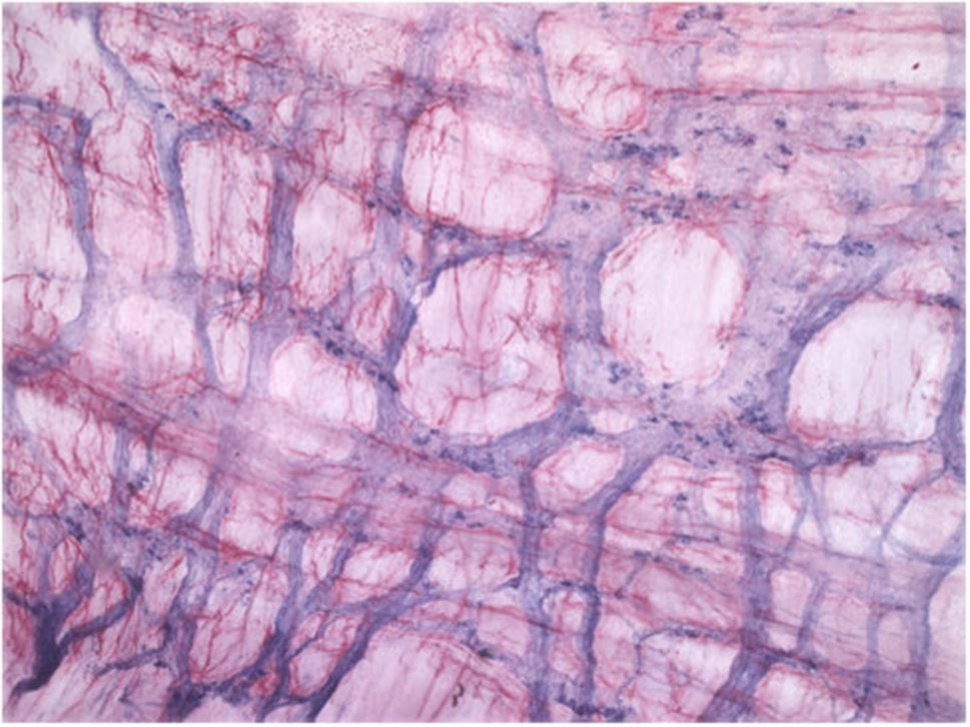
Whole-mount preparation of longitudinal muscle of human colon, myenteric plexus stained with NADPH-diaphorase (blue) and myenteric ICCs stained with anti-c-kit immunohistochemistry (red)

**Fig. 3:**
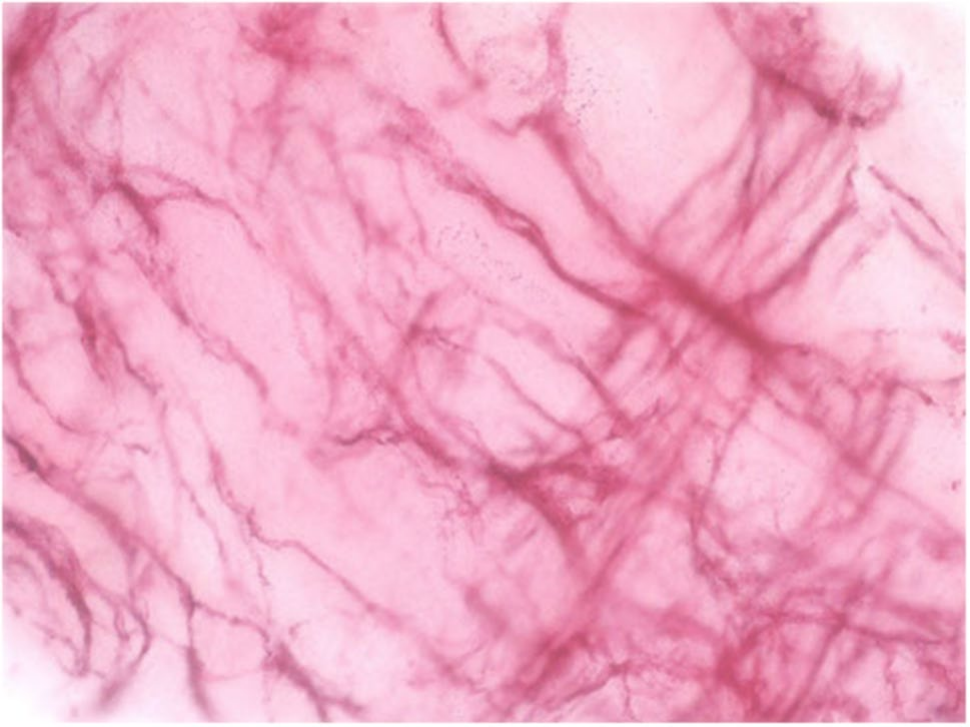
Whole-mount preparation of longitudinal muscle of human colon, nerve fibers stained with NADPH-diaphorase (blue) and muscular ICCs stained with anti-c-kit immunohistochemistry (red)

Over the years, the distribution and morphology of ICCs have been analyzed by many methods. Historically, traditional histology stains such as methylene blue, silver or Golgi impregnation were used. These specific staining techniques led to the previous assumption that ICCs are primitive neurons, as they were unable to truly discriminate between neurons and ICCs. Later, electron microscopy was applied to further enable ultrastructural studies of ICCs [45–48]. To date, electron microscopy remains the method of choice for the examination of the typical ultrastructural features of ICCs, including their well-developed smooth endoplasmic reticulum, abundant intermediate filaments, lack of myosin filaments, numerous caveolae, dense bodies and bands as well as an oval indented nucleus [49–57]. Furthermore, ICCs are intercalated between neurons and smooth muscle cells and have been shown to form gap junctions with the latter cell type [58]. In addition to the characterization of single cells, the distribution and topography of ICCs in various tissues have been investigated in great detail. The identification of c-kit expression in ICCs was a major scientific breakthrough. This proto-oncogene that encodes the receptor tyrosine kinase kit is highly expressed in both ICCs and mast cells [7, 30, 31, 59, 60]. Consequently, many studies have been performed showing the expression of c-kit-positive ICCs in the gastrointestinal tract of several species, including humans [10, 26, 61, 62], mice [26], rats [63, 64], and guinea-pigs [65, 66]. These studies and further investigations have increased our understanding of the complex architecture of ICC networks in relation to the ENS and the intestinal smooth muscle layers [67]. Recently, Ano1 has been identified as a highly specific marker for all subtypes of ICCs within the gastrointestinal tract of mice and humans, and its expression has been associated with the generation of electric slow waves [12] (Figs. 1, 2 and 3).

**Fig. 4:**
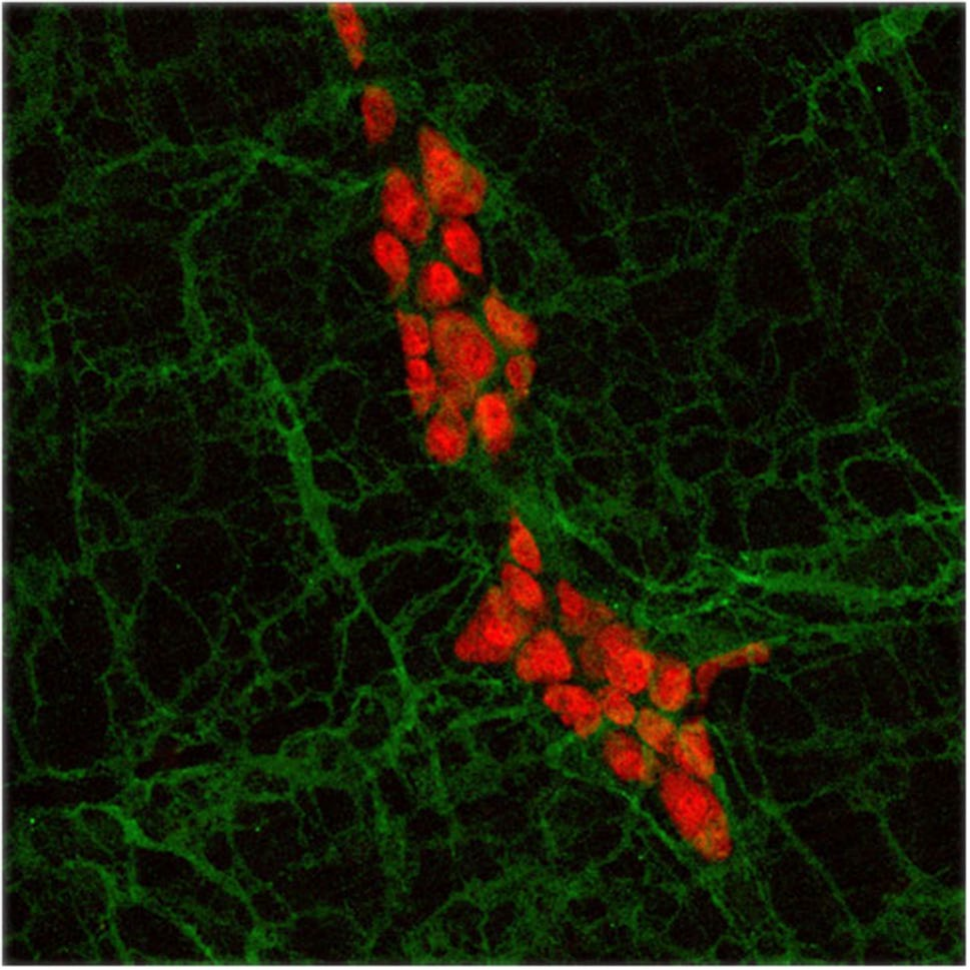
Whole-mount preparation of mouse small bowel, myenteric plexus stained with anti-hu-immunohistochemistry (red) and myenteric ICC stained with anti-c-kit-immunohistochemistry (green)

The complex relationship of ICCs to the ENS and other surrounding structures was traditionally investigated in conventional thin histological sections. The development of whole-mount preparation has proven to be an effective technique for the visualization of the structure of the intrinsic networks (e.g., neurons and ICCs) and their patterns of branching and interconnection with each other and neighboring tissue layers (Fig. 4). It facilitates the three-dimensional study of the morphology of neuronal and ICC networks [68, 69], having some obvious advantages compared to conventional histological thin sections, as it enables a more detailed examination of the complex morphology of neurons, glial cells or ICC within the gut [70]. Whole-mount preparations can comprise several layers of bowel wall, including the longitudinal muscle layer and the adjacent myenteric plexus. They are made by separating the muscular layer from the submucosal layer, followed by removal of the circular muscle layer from the longitudinal muscle. Subsequently, the mucosa is removed from the submucosal layer to better visualize the submucosal plexus. The three-dimensional configuration of c-kit-positive cells was first described as typical for multipolar cells around the myenteric plexus and slender bipolar cells within the circular and longitudinal muscle layers [61]. Furthermore, close relationships between muscular ICCs and neurons with nitric-oxide synthase-like immunoreactivity, vesicular acetylcholine transporter and substance P-like immunoreactive axonal varicosities have been demonstrated in whole-mount preparations of guinea-pig small intestine [71]. Therefore, it has been assumed that enteric motor neurons, ICC, and smooth muscle cells form functional units [71, 72]. The close connections between ICCs and the intrinsic nitrergic innervation (e.g., NADPH-diaphorase-positive nerve fibers) have been shown in the human gut [68]. Several investigators have used the whole-mount preparation technique in specimens from the human gastrointestinal tract in combination with various other ENS staining methods, ranging from silver impregnation to enzyme histochemistry and immunohistochemistry [73–75].

### ICCs in gastrointestinal motility disorders in childhood

Gastrointestinal motility disorders describe a heterogeneous group of conditions in infants and young children in which symptoms of functional bowel obstruction arise from a neuromuscular dysfunction of the colon and rectum (including ICCs or glial cells) [76]. In many cases, the exact pathogenesis remains poorly understood. There are a number of patients that present with clinical symptoms similar to Hirschsprung disease (HD) despite the presence of ganglion cells in rectal biopsies. Over the years, various terms such as “*chronic idiopathic intestinal pseudo-obstruction*”, “*intestinal hypoperistalsis syndrome*” or “*pseudo-HD*” have been used to describe these gastrointestinal motility disorders. In 1997, the pediatric surgeon Prem Puri suggested that “*variant HD*” may be a more appropriate description [77]. C-kit labeling has been used to study the pathological variations of ICCs in these conditions and absence or deficiency of ICC networks was identified.

#### Hirschsprung disease

**Fig. 5:**
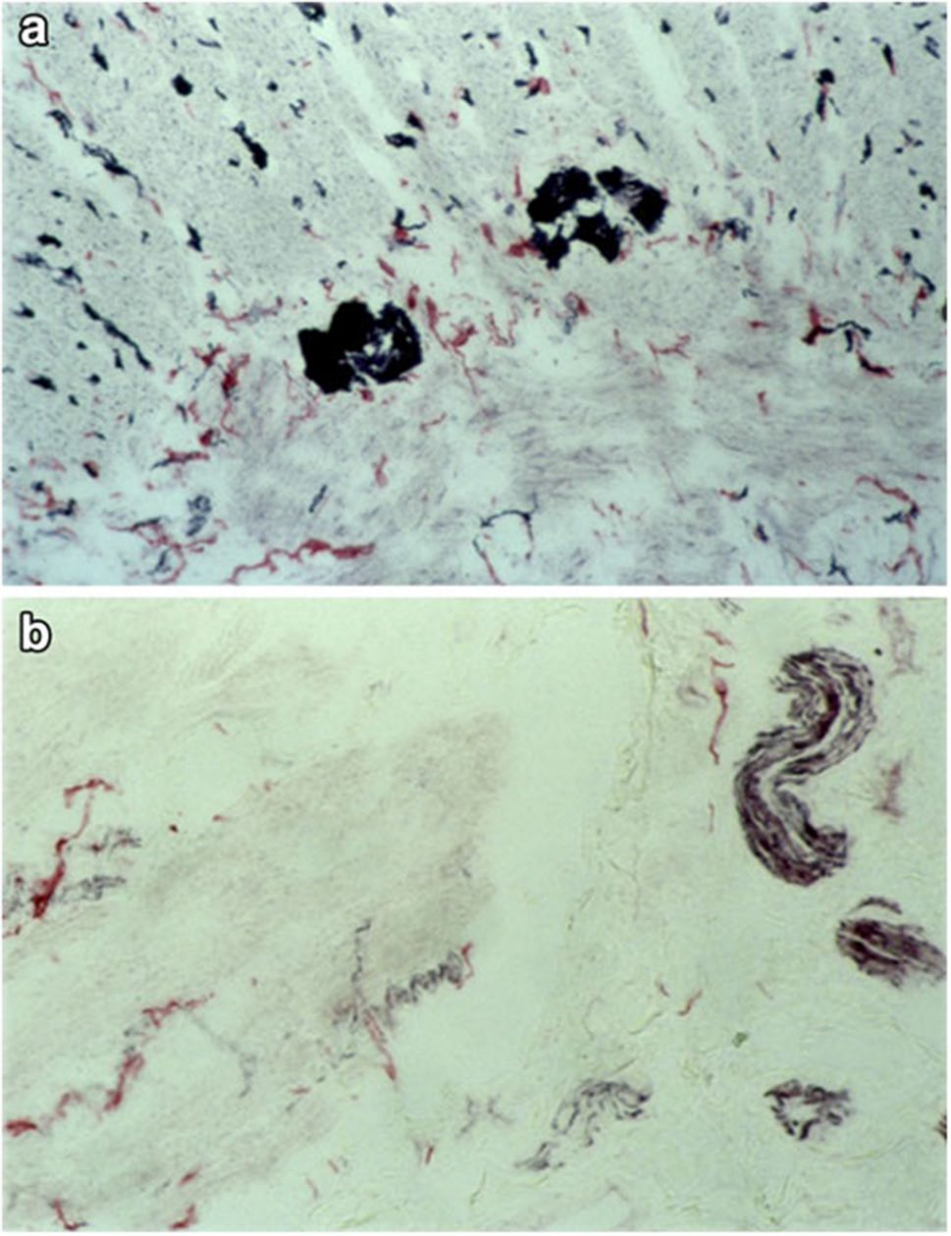
Section of normal bowel (**a**) and HD bowel (**b**) stained with NADPH-diaphorase and anti-c-kit-immunohistochemistry

HD is one of the most common congenital gastrointestinal motility disorders. Clinical symptoms related to functional bowel obstruction, such as delayed first passage of meconium, abdominal distension, and bilious vomiting result from a congenital aganglionosis in the most distal part of the gastrointestinal tract. The distribution of ICCs has widely been studied in HD bowel using normal histology sections and whole-mount preparation techniques. The focus of these investigations has not been restricted to the aganglionic segment but has extended to the ganglionic segment in HD [61, 72, 78–84]. Most of these studies have shown a reduced number of c-kit-positive ICCs in the aganglionic bowel and also in the transition zone of HD patients [72, 78–84]. Major pathological features in HD included a reduced number of myenteric ICCs and disrupted myenteric ICC networks that are only sparsely distributed between hypertrophic nerve trunks (Fig. 5). Furthermore, muscular ICCs have been found to be markedly reduced in the HD bowel [79, 80]. Interestingly, a total reduction of ICCs in the proximal, ganglionic colon of HD patients has also been observed in comparison to healthy controls [85]. This observation has been contested by others, who did not find an overall difference in the distribution of c-kit-positive ICCs [80, 84, 86, 87]. However, a marked variability of ICC values in patients with HD has been noted, which may be a reflection of the heterogeneous character of this disease [86]. In addition, two studies linked poor clinical outcomes in HD patients to very low numbers of ICCs and a low ratio of ICCs to neural innervation [86, 88]. More recently, the use of c-kit has been replaced by the more specific ICC marker Ano1, showing a moderate reduction of ICC fibers in ganglionic HD colon, compared to the colon of non-HD patients [89]. These contradicting results are likely to arise from small study populations, but may have also been biased by the fact that the distribution of ICCs can vary with age and location in the gastrointestinal tract [8, 85, 89]. Moreover, an important question is whether the observed alterations of ICCs in HD are truly primary or whether they are secondary to long-lasting functional obstruction. Therefore, the role of structural ICC abnormalities in persistent bowel dysfunction in patients with HD is still to be elucidated.

#### Intestinal neuronal dysplasia

The pathologist William A. Meier-Ruge described intestinal neuronal dysplasia (IND) in 1971 as a hyperplastic malformation of the enteric plexus [90]. A few years later, a case of rectosigmoid aganglionosis associated with IND of the descending and transverse colon has been reported [91]. Nowadays, IND can be classified into two clinical and histological distinct subtypes [92]: IND type A (IND A), occurring in less than 5% of all IND cases, is characterized by congenital aplasia or hypoplasia of the sympathetic innervation. Normally, patients with IND A present in the neonatal period with episodes of abdominal distension, intestinal obstruction and diarrhea with bloody stools. Conversely, IND type B (IND B) is defined by hyperplasia of the parasympathetic submucosal and myenteric plexuses, accounting for over 95% of all IND cases. Typical histological features of IND B include hyperganglionosis, giant ganglia, ectopic ganglion cells, and increased activity of acetylcholinesterase (AChE) in the lamina propria and around submucosal blood vessels [93]. IND occurring in association with HD is invariably IND B. While some authors have found IND in up to 44% of their HD patients, others have rarely encountered IND in association with HD [94]. The existence of IND as a distinct histopathological entity remains controversial [95]. Hence, several researchers have suggested that the observed changes in IND may be either a variant of normal bowel development or a secondary acquired phenomenon caused by congenital obstruction or inflammation [96]. Nevertheless, a reduced number of c-kit-positive ICCs has been demonstrated in the myenteric plexus and muscle layers of IND cases [97].

#### Isolated hypoganglionosis

Isolated hypoganglionosis (HG) is a rare entity, which has been classified as a hypogenetic type of intestinal innervation disorders. The clinical presentation of patients with isolated HG is similar to those with classical HD with non-specific symptoms of severe constipation or bowel obstruction. It has been shown that congenital and acquired HG are two separate entities with different clinical features and histological findings [98]. At present, there are only a few cases in the published literature as isolated HG is one of the rarest types of gastrointestinal motility disorders and there remains controversy regarding it as a distinct isolated histopathological entity [96]. Some cases of isolated HG were reported to exhibit deficient expression of c-kit-positive ICCs within the myenteric plexus and the smooth muscle layer, which may contribute to the observed motility dysfunction in the hypoganglionic bowel segment. C-kit staining has been employed to investigate the expression of ICCs and, thus, intestinal pacemaker activity, which is markedly decreased or even absent in patients with isolated HG [99].

#### Internal anal sphincter achalasia

Internal anal sphincter achalasia (IASA) has a similar clinical presentation to HD, but with the presence of ganglion cells in rectal biopsies. Previously, IASA was referred to as ultrashort-segment HD, which is characterized by an aganglionic segment of 1–3 cm above the pectinate line, normal AChE activity in the lamina propria and increased AChE activity in the muscularis mucosae [100]. Thus, it has been suggested that IASA is a more accurate term for this pathological entity as many patients with absence of the rectosphincteric reflex on anorectal manometry actually showed presence of ganglion cells combined with normal AChE activity in rectal biopsies [101]. Despite attempts of numerous investigators to determine the pathophysiological mechanisms of IASA in more detail, the exact pathogenesis remains unknown. Age-related changes in the developing intramuscular innervation of the internal anal sphincter (IAS) most likely form the basis for the observed motility dysfunction [101]. Additionally, a reduced number of c-kit-positive ICCs has been found in the IAS of patients with IASA [97]. The deficiency in nitrergic innervation and ICCs may explain the impaired IAS relaxation in these cases.

#### Megacystis microcolon intestinal hypoperistalsis syndrome

Megacystis microcolon intestinal hypoperistalsis syndrome (MMIHS) is an extremely rare condition and the most severe form of functional bowel obstruction in the newborn, characterized by massive abdominal distension caused by a large-dilated non-obstructed bladder, microcolon with malrotation and decreased or absent intestinal peristalsis [102]. MMIHS was first observed in 1976 in five newborn girls [103]. Since then, various hypotheses have been proposed to explain the pathogenesis of MMIHS. Genetic, myogenic, neurogenic, and hormonal etiologies have been discussed. However, most of these theories were derived from case reports due to the rarity of this condition. Thus, the etiology remains poorly understood. Histological evaluation of myenteric and submucosal plexuses has revealed normal ganglion cells in 77% of the investigated bowel specimens from patients with MMIHS. The remaining 23% were shown to have various neuronal abnormalities including hyper-/hypoganglionosis and immature ganglia [100]. Furthermore, some authors found significant anomalies in smooth muscle cells from bowel and bladder specimens, such as vacuolar degeneration as well as thinning of the longitudinal muscle [104, 105]. Likewise, a decreased expression of ICCs in the bladder has been observed [97].

## Conclusion and future directions

ICCs have a central function in the generation and propagation of gastrointestinal slow-wave activity and their loss might result in gastrointestinal motility dysfunction. Most of the available research studies have shown that HD and allied disorders are associated with either a loss of or deficiency in ICC networks. However, these findings require careful interpretation, as our current understanding of the nature of the relationships between the loss of ICCs and the development of clinical symptoms in humans is incomplete. It should be noted that all investigated specimens had previously been subjected to long-lasting functional obstruction. Thus, it is difficult to determine whether the loss or deficiency of ICCs is the consequence or the cause of the disease process. Consequently, further clinical and animal studies are necessary to improve our knowledge regarding the true importance of the impaired ICC function in infants and children with gastrointestinal motility disorders.
